# Oxaliplatin Analogues with Carboxy Derivatives of Boldine with Enhanced Antioxidant Activity

**DOI:** 10.1155/2015/920143

**Published:** 2015-02-28

**Authors:** Marco Mellado, Carlos Jara, David Astudillo, Joan Villena, Patricio G. Reveco, Franz A. Thomet

**Affiliations:** ^1^Departamento de Química, Universidad Técnica Federico Santa María, Avenida España 1680, 2390123 Valparaíso, Chile; ^2^Centro de Investigaciones Biomédicas (CIB), Escuela de Medicina, Universidad de Valparaiso, Avenida Hontaneda 2664, 2340000 Valparaíso, Chile; ^3^Laboratorio de Investigación-Estrés Oxidativo, Escuela de Medicina, Universidad de Valparaiso, Avenida Brasil 1560, 2340000 Valparaíso, Chile

## Abstract

A new oxaliplatin analog [Pt(dach)(L5)] (**1**) was synthesized and characterized as a continuation of a study of the previously reported [Pt(dach)(L6)] (**2**), where dach = (1*R*,2*R*)-diaminocyclohexane, L5 = 3-carboxyboldine, and L6 = 3-carboxypredicentrine. Compounds **1** and **2** exhibited a substantially enhanced antioxidant activity compared to oxaliplatin (130 and 30 times for **1** and 13 and 4 times for **2** using the DPPH and FRAP assays, resp.). In addition, **1** and **2** exhibited cytotoxic activity in the same range as oxaliplatin toward the two human tumor cell lines (MCF-7 and HT-29) studied and two to four times lower activity in the human colon nontumor cell line (CCD-841). Preadministration of L5 or L6 to the colon tumor (HT-29) and the colon nontumor (CCD-841) cell lines prior to oxaliplatin addition increased the viability of the nontumor cell line to a greater extent than that of the tumor cell line.

## 1. Introduction

The discovery of the biological properties of cisplatin has been the most relevant event in metal-based chemotherapeutics to date. The worldwide use of cisplatin over the last 35 years for different types of cancer (i.e., testicular, ovarian, and bladder cancer) has been limited due to its deleterious side effects, such nephro-, hepato-, cardio-, and ototoxicity, as well as its chemical resistance [1]. The development of second and third generation drugs (i.e., carboplatin and oxaliplatin, resp.) has partially reduced these drawbacks. However, myelosuppression for carboplatin and neurotoxicity for oxaliplatin remain the primary secondary toxicities [2]. To decrease these undesired effects and/or to circumvent the cisplatin resistance, different strategies have been developed to design of new compounds [3]. Promising developments include the synthesis of picoplatin, which is a sterically hindered 2-methylpyridine platinum(II) compound with reduced reactivity in sulfur ligand reactions [4], and satraplatin, which is a platinum(IV) compound that releases an active platinum(II) metabolite after intracellular reduction [5]. Biologically relevant molecules, such as estrogenic steroids, folic acid, sugar derivatives, antitumor natural products, and platinum-resistance modulators, have been coordinated to Pt(II) and Pt(IV) moieties to target tumor tissues [6–10]. The design of an improved drug delivery system is another field of chemotherapeutics that aims to provide treatment with fewer undesired effects. Liposomes, dendrimers, and carbon nanotubes have received increased attention in the last few years due to their potential for improving drug efficacy and reducing undesired secondary toxicities [11].

In recent years, increasing evidence suggests that a combination therapy involving natural chemopreventive agents and platinum-based drugs can enhance the effectiveness of the treatment [12] or reduce some of the metal induced secondary toxicities [13]. Quercetin, resveratrol, anethole, and curcumin have exhibited a synergic effect in combination with cisplatin and oxaliplatin in an* in vitro* model [14–16]. However, curcumin and capsaicin have exhibited a protective effect against cisplatin induced nephro- and neurotoxicity [17, 18].

To explore the potential pharmacological synergism between metal-based drugs and natural products, our research group has focused on the synthesis of these types of compounds employing natural derivatives as ligands [19, 20]. Boldine (L1) is the main constituent of the endemic Chilean boldo tree (*Peumus boldus* Molina, Monimiaceae) and possesses important health-promoting properties, such as antioxidant, anti-inflammatory, and cytoprotective effects [21]. The use of boldine in a pretreatment regime decreased the cisplatin-induced lipoperoxidation in mice liver [22]. [Pt(dach)(L6)] (**2**; where dach = (1*R*,2*R*)-diaminocyclohexane and L6 = 3-carboxypredicentrine) exhibited comparable cytotoxic activity toward three human tumor cell lines and a tenfold smaller biological activity in a nontumor cell line than the commercial drug oxaliplatin [19]. Herein, we described the synthesis of 3-carboxyboldine (L5) and the corresponding platinum(II) compound [Pt(dach)(L5)] (**1**). The antioxidant activity of L5 and L6 (as chlorohydrate salts) and metal complexes** 1** and** 2** was evaluated based on two chemical-based models (DPPH and FRAP assays), and the* in vitro* cytotoxic activity was evaluated for two human tumor cell lines (MCF-7 and HT-29) and one nontumor cell line (CCD-841). Also, to evaluate the potential cytoprotecting effect of the carboxy derivatives, especially on a nontumor cell line, pretreatment experiments using L5 or L6 (as chlorohydrate salts) with oxaliplatin on a human colon tumor cell line (HT-29) and a human nontumor cell line (CCD-841) were performed.

## 2. Materials and Methods

### 2.1. General Considerations

The NMR experiments were performed using an Avance 400 Digital Bruker NMR spectrometer operating at 400.13 MHz for ^1^H and at 100.61 MHz for ^13^C. The chemical shifts (*δ*) are given in ppm, and the coupling constant (J) is reported in Hz. The ^1^H NMR chemical shifts are reported relative to the proton signal of incompletely deuterated DMSO-d_6_ (*δ* 2.49), and the ^13^C NMR chemical shifts are relative to the carbon of DMSO-d_6_ (*δ* 39.5). The peak assignments were further confirmed by 2D-HSQC/HMBC experiments. The mass spectra were recorded on an ESI-IT Esquire 4000 Bruker spectrometer by direct injection using the Esquire Control 5.2 software. The infrared spectra were recorded on a FTIR Nicolet 4600 instrument, and the elemental analyses were performed on a Flash EA 1112.

Boldine (L1) was donated by INDENA. K_2_PtCl_4_, dach, KI, Ag_2_SO_4_, AgNO_3_, and Ba(OH)_2_  ×  H_2_O were purchased from Aldrich. 2,9-Dimethoxymethyl-3-bromoboldine (L3) was prepared according to a previously published protocol with some modifications [23]. Chloromethyl methyl ether was synthesized using the method reported by Marvel and Porter [24], and the Pt complex [Pt(dach)I_2_] was prepared from K_2_PtCl_4_ according to the procedure reported by Khokhar et al. [25]. 3-Carboxypredicentrine·HCl (L6) and boldiplatin (**2**) were prepared according to a previously published protocol [19]. The anhydrous solvents (i.e., acetone (K_2_CO_3_), pyridine (4A molecular sieves), and THF (Na/benzophenone)) were dried and freshly distilled. All of the other reagents were obtained from commercial suppliers and used without further purification.

### 2.2. Synthesis of 3-Carboxyboldine (L5·HCl)

To a solution of 1.15 g of 2,9-dimethoxymethyl-3-bromoboldine (2.3 mmol) in 40 mL of dry THF at −78°C, 3.0 mL of n-BuLi 1.6 M in hexane (4.8 mmol) was added dropwise. After 1 minute, the cooling bath was removed, and CO_2_ gas was introduced into the solution for 10 minutes. The mixture was stirred for 2 hours at room temperature. The solvent was removed under reduced pressure, and the solid was dissolved in an acid mixture (12 mL isopropanol, 12 mL THF, and 5 mL concentrated HCl). The solution was stirred overnight at room temperature. The acid medium was removed under reduced pressure, and the residue was purified by column chromatography (0.040–0.063 mm) employing an increasingly polar mixture of CHCl_3_ : MeOH = 9 : 1 → 1 : 1 as the eluent. Yield: 0.461 g (1.1 mmol, 48%) white solid. m.p. > 170°C (dec). ^1^H NMR (400 MHz, DMSO-d_6_, 25°C) *δ* 2.40 (1H, dd, J = 13.4 and 13.6 Hz, H-7), 2.64 (3H, s, N-CH_3_), 3.02 (1H, d, J = 12.6 Hz, H-7), 3.1–3.3 (2H, m, H-4 + H-5), 3.58 (1H, d, 16.8 Hz, H-4), 3.64 (3H, s, 1-OCH_3_), 3.77 (3H, s, 10-OCH_3_), 6.72 (1H, s, H-8), 7.85 (1H, s, H-11), 9.24 (1H, s, OH). ^13^C NMR (100 MHz, DMSO-d_6_, 25°C) *δ* 27.4 (C-4), 32.4 (C-7), 42.2 (N-CH_3_), 52.4 (C-5), 55.7 (10-OCH_3_), 58.5 (1-OCH_3_), 62.5 (C-6a), 112.8 (C-11), 114.9 (C-8), 116.4 (C-3), 122.6 (C-11a), 127.2, 128.8, 129.3, 143.3 (C-1), 146.0, 146.1 (C-10), 157.8, 172.0 (COOH). IR (KBr disk) 3432 *υ*(O-H), 2961, 2934, 1613 *υ*(C=O), 1572, 1510 cm^−1^. ESI-MS (positive mode) *m*/*z* 372 [MH-Cl]^+^. Anal. Calc. for C_20_H_22_NO_6_Cl: C, 58.90; H, 5.44; N, 3.43. Found: C, 59.35; H, 5.60; N, 3.60.

### 2.3. Synthesis of [Pt(dach)(L5)] (**1**)

A mixture of 0.340 g of [Pt(dach)I_2_] (0.60 mmol) and 0.206 g of AgNO_3_ (1.20 mmol) in 20 mL of deionized water was stirred for 24 hours in the dark at room temperature. The AgI precipitate was removed by filtration, and the filtrate was reacted with a freshly prepared solution of barium 3-carboxyboldinate (prepared from 0.240 g, 0.59 mmol of 3-carboxyboldine with 0.115 g, 0.60 mmol of Ba(OH)_2_  ×  H_2_O in 5 mL of deionized water) for 24 hours at room temperature. The precipitate was filtered, washed with deionized water (2 × 3 mL), and dried. Yield: 0.186 g (0.27 mmol, 46% based on Pt complex). ^1^H NMR (400 MHz, DMSO-d_6_, 25°C) *δ* 0.98 (br s, 2H, H-*χ*), 1.23 (br s, 2H, H-*β*), 1.44 (br s, 2H, H-*χ*), 1.90 (br s, 2H, H-*β*), 2.10–2.25 (2H, m, H-5 + H-7), 2.33 (3H, s, N-CH_3_), 2.68 (1H, d, J = 14.2 Hz, H-6a), 2.80–2.95 (2H, m, H-5 + H-7), 3.60 (3H, s, 1-OCH_3_), 3.76 (3H, s, 10-OCH_3_), 6.68 (1H, s, H-8), 7.84 (1H, s, H-11), 9.12 (1H, s, OH). ^13^C NMR (100 MHz, DMSO-d_6_, 25°C) *δ* 24.2 (C-*χ*), 29.3 (C-4), 31.6 (C-*β*), 34.0 (C-7), 44.1 (N-CH_3_), 53.3 (C-5), 55.8 (10-OCH_3_), 58.6 (1-OCH_3_), 63.6 (C-6a), 112.8 (C-11), 114.8 (C-8), 116.6 (C-3), 122.8, 122.9, 127.5 (C-1a), 130.4, 130.9, 142.8 (C-1), 145.8, 146.0 (C-10), 156.3, 172.9 (COO). IR (KBr disk) 3447 *υ*(O-H), 3238 *υ*(N-H), 2934, 1607 *υ*(C=O), 1569, 1507 cm^−1^. ESI-MS (positive mode) *m*/*z* 679 [MH]^+^. Anal. Calc. for C_26_H_33_N_3_O_6_Pt: C, 46.02; H, 4.90; N, 6.19. Found: C, 46.55; H, 4.70; N, 6.05.

### 2.4. DPPH Assay

The DPPH assay was performed using a previously described method [26, 27]. 0.1 mL of compounds L1, L5, L6,** 1,** and** 2** (from 0.20 to 10 mM solutions in ethanol) were mixed with 2.9 mL of a freshly prepared solution of DPPH^*∙*^ (2,2-diphenyl-1-picrylhydrazyl) in ethanol (50 *μ*M). A solution obtained by mixing 2.9 mL of the DPPH^*∙*^ solution with 0.1 mL of ethanol was used as the control. The absorbance of the resulting solutions, control, and blank (reagents only) was recorded after 15 min at room temperature. Each sample was replicated three times. The disappearance of DPPH^*∙*^ was detected spectrophotometrically at 517 nm. The percent radical scavenging capacity (RSC) was calculated according to the following equation:

(1) $$\begin{array}{c} \text{RSC}\left(\%\right)=100\%\times \frac{\left(A_{\text{control}}-A_{\text{sample}}\right)}{A_{\text{control}}}. \end{array}$$

From the obtained RSC(%) values, the IC_50_ value, which represents the concentration of compounds that results in 50% neutralization, was determined by linear regression analysis.

### 2.5. FRAP (Ferric Reducing Antioxidant Power) Assay

The ferric reducing power of compounds L1, L5, L6,** 1,** and** 2** was measured according to a previously published protocol with modifications [28]. This method is based on the reduction of a colorless ferric complex (Fe^3+^-tripyridyltriazine) at a low pH to a blue-colored ferrous complex (Fe^2+^-tripyridyltriazine) due to electron-donating antioxidants. The reduction was monitored by measuring the change in the absorbance at 593 nm. The working FRAP reagent was prepared daily by mixing 10 volumes of a 300 mM acetate buffer at pH 3.6 with 1 volume of 10 mM TPTZ (2,4,6-tri(2-pyridyl)-*s*-triazine) in 40 mM hydrochloric acid and 1 volume of 20 mM ferric chloride. A standard curve was prepared using various concentrations of Trolox. All of the solutions were prepared daily. 100 *μ*L of the sample solutions and 300 *μ*L of deionized water were added to 3 mL of the freshly prepared FRAP reagent. The reaction mixture was incubated for 30 min at 37°C in a water bath. Then, the absorbance of the samples was measured at 593 nm. A sample blank reading using ethanol was also recorded. The difference between the sample absorbance and the blank absorbance was determined and used to calculate the FRAP value. In this assay, the reducing capacity of the tested compounds was calculated in reference to the reaction signal given by a Trolox solution. The FRAP values are expressed as mM Trolox. All of the measurements were performed in triplicate.

### 2.6. *In Vitro* Inhibition Growth Assay

The experimental cell cultures were obtained from the American Type Culture Collection (Rockville, MD, USA). The HT-29 colon cancer cell line, MCF-7 breast adenocarcinoma cell line, and CCD-841 (CoN) human colon epithelial cell line were grown in Dulbecco's modified Eagle's medium (DMEM) containing 10% FCS, 100 U/mL penicillin, 100 *μ*g/mL streptomycin, and 1 mM glutamine. The cells were seeded into 96-well microtiter plates in 100 *μ*L volumes at a plating density of 5 × 10^3^ cells/well. After 24 h of incubation at 37°C under a humidified 5% CO_2_ atmosphere to allow for cell attachment, the cells were treated with different concentrations of the drugs (L5, L6,** 1**,** 2,** and oxaliplatin) and incubated for 72 h under the same conditions. The sulforhodamine B assay was used to evaluate cell viability according to the method reported by Skehan et al. [29, 30]. Next, the cells were fixed with 50% trichloroacetic acid at 4°C. After washing with water, the cells were stained with 0.1% sulforhodamine B (Sigma-Aldrich, St. Louis, MO, USA), dissolved in 1% acetic acid (50 *μ*L/well) for 30 min and subsequently washed with 1% acetic acid to remove any unbound stain. The protein-bound stain was solubilized with 100 *μ*L of 10 mM unbuffered Tris base, and the cell density was determined using a spectrophotometric plate reader (wavelength 540 nm). The values are reported as the means ± SD of three independent experiments. The GraphPad software (GraphPad Software, San Diego, CA, USA) was used to calculate the IC_50_ values. For the preadministration of 3-carboxyboldine (L5·HCl) or 3-carboxypredicentrine (L6·HCl), the cells were previously pretreated for 24 h prior to oxaliplatin addition. The stock solutions of the compounds were prepared in DMSO, and the final concentration of this solvent was maintained at 0.1%. The control cultures were only treated with 0.1% DMSO.

### 2.7. Statistical Analysis

The data in Sections 2.4 and 2.5 were reported as mean values ± standard deviation (SD). After tests of normality and homoscedasticity (Kolmogorov–Smirnov and Cochran tests, resp.), Kruskal-Wallis ANOVA was used with a confidence level of 95%. The values that represent the concentrations of investigated compounds that cause 50% inhibition (IC_50_) were determined by linear regression analysis of the radical scavenging capacity (% RSC). A similar procedure was performed for the FRAP assays (STATISTICA 7.0 program).

## 3. Results and Discussion

### 3.1. Synthesis of 3-Carboxyboldine (L5·HCl)

**Scheme 1:**
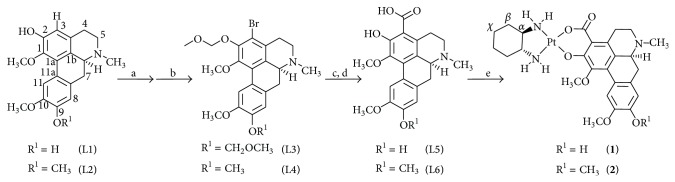
General synthetic procedure for compounds L5, L6, **1** and **2**. a: NBS (1.1 equiv), CF_3_COOH, room temperature, 2 h; b: NaOH, H_2_O, CH_3_OCH_2_Cl, anhydrous acetone, anhydrous pyridine, room temperature, 2 h; c: n-BuLi 1.6 M, anhydrous THF, −78°C → room temperature, CO_2_ (10 min.); d: HCl 2M, i-PrOH, THF, room temperature, 16 h; e: [Pt(dach)(OH_2_)_2_](NO_3_)_2_ or [Pt(dach)(OH_2_)(OSO_3_)], H_2_O, room temperature, 16 h.

3-Carboxyboldine was obtained employing the lithium-bromide exchange reaction strategy under the same experimental conditions reported for 3-carboxypredicentrine (L6·HCl) [19]. The lithiation precursor 2,9-dimethoxymethyl-3-bromoboldine (L3) was prepared as described in the literature [23]. However, the use of dry acetone instead of dry ethanol improved the methoxymethylation yield from 24 to 52%.  Scheme 1 summarizes the synthetic procedure performed where L5 was isolated as a chlorohydrate salt. However, better ^1^H and ^13^C NMR spectra were recorded for L5 in the free base form. The ^1^H NMR spectra contain the signals associated with the three characteristic methyl groups at *δ* 2.64 (N-CH_3_), 3.64 (1-OCH_3_), and 3.77 (10-OCH_3_) as well as the two aromatic protons at *δ* 6.72 (H-8) and 7.85 (H-11) ppm. The proton spectra also revealed one phenolic proton at 9.24 ppm in DMSO-d_6_ (and in CD_3_OD) and no additional signals associated with the second OH and COOH proton at lower fields. For the previously reported L6·HCl, no signals associated with these functional groups were observed [19]. The ^13^C NMR spectra of 3-carboxyboldine (L5) contained a signal at 172.0 ppm (associated to COOH) in addition to the characteristic signals of the natural precursor, and most of these signals were assigned by 2D-HSQC/HMBC experiments. The ESI mass spectrum (positive mode) contained the *m*/*z* 372 [M+H-Cl]^+^ fragments, and the negative mode spectrum revealed the *m*/*z* 370 [M-H-Cl]^−^ fragment, confirming the presence of a compound with the general formula C_20_H_21_NO_6_ (molecular weight 371 g/mol). The characterization of compound L5 was also confirmed by reaction with freshly prepared CH_2_N_2_ to form the methylated derivative. Thin layer chromatography analysis and the proton and carbon chemical shifts of the methylated derivative are in agreement with the signals of previously reported 3-carboxypredicentrine (L6).

### 3.2. Synthesis of [Pt(dach)(L5)] (**1**)

An aqueous solution of [Pt(dach)(OH_2_)_2_](NO_3_)_2_ was obtained by reaction of [Pt(dach)I_2_] with 2 equivalents of AgNO_3_ for 24 hours in the absence of light. This solution was reacted with a freshly prepared barium 3-carboxyboldinate solution to yield** 1** as a purple insoluble product in 46% yield. The ^1^H NMR chemical shifts were compared with those previously reported for [Pt(dach)(L6)] (**2**), and the expected differences were observed as follows: (a) an additional signal at *δ* 9.12 (1H, s) associated with the phenol at position C-9 and (b) the disappearance of one signal at approximately *δ* 3.7 (3H, s) associated with the OCH_3_ group at C-9 position. These results confirmed the coordination of 3-carboxyboldine (see Supplementary Material available online at http://dx.doi.org/10.1155/2015/920143). The ^13^C NMR spectra show the carbonyl chemical shift at *δ* 172.9 ppm (in agreement with *δ* 172.3 for** 2**), and most of the signals were assigned by 2D-HSQC/HMBC experiments. The infrared spectrum showed the partial disappearance of the broad band from 2500 to 3500 cm^−1^ (including carboxylic acid and phenol OH stretching) along with a slight shift in the CO band from 1613 cm^−1^ in L5 to 1607 cm^−1^ in** 1**. In addition, a new band at 3238 cm^−1^ associated with N-H stretching in** 1** was observed (see Supplementary Material). The ESI mass spectrum (positive mode) shows the parent ion at *m*/*z* 679 (MH^+^), and the ions at *m*/*z* 372 and *m*/*z* 328 are associated with the protonated form of 3-carboxyboldine and boldine, respectively.

### 3.3. DPPH Assay

The antioxidant activity of boldine (L1) and the 3-carboxyboldine (L5·HCl) and 3-carboxypredicentrine (L6·HCl) synthesized derivatives was investigated to compare the radical scavenger properties of the derivatives with the natural precursor. In addition, platinum(II) coordination compounds** 1** and** 2** were also tested to evaluate their antioxidant activity upon boldine derivative coordination.  Table 1 shows the antioxidant activity (expressed as IC_50_ values) of these compounds in the DPPH assay. For comparison, Table 1 also includes the results for the commercial drug oxaliplatin and the commercial antioxidant Trolox.

Based on the results, 3-carboxyboldine (L5) exhibits a scavenging activity toward stable free radicals that is 18 times greater than that of 3-carboxypredicentrine (L6) and approximately half of the activity exhibited by the natural precursor boldine (L1). This result suggests that the introduction of the carboxy substituent at the C-3 position of boldine does not favor the H• donor ability of the compounds. The presence of the phenol at position C-9 in 3-carboxyboldine (L5) contributes to its higher antioxidant activity. However, platinum coordinated compounds** 1** and** 2** exhibited a considerably higher radical scavenger behavior (130 and 13 times, resp.) than the commercial drug oxaliplatin and even better than the corresponding carboxy derivatives (i.e., L5 and L6). The IC_50_ values obtained for** 1** and** 2** in this chemical assay indicate that coordination of antioxidant compounds L5 and L6 to the Pt(dach) moiety substantially enhances the antioxidant behavior of the oxaliplatin analogues.

### 3.4. FRAP Assay

**Figure 1:**
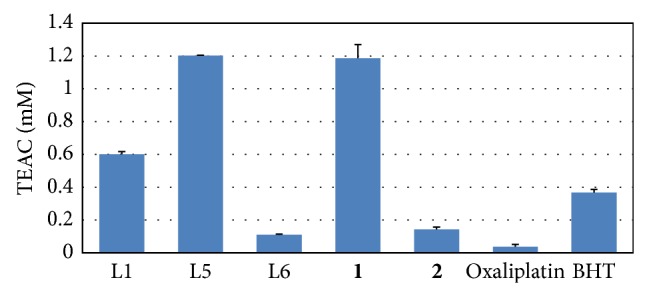
Antioxidant activity (Trolox equivalent activity capacity, TEAC) at 1 mM concentration, in FRAP assay.

Figure 1 shows the antioxidant activity (expressed as Trolox equivalent activity capacity, TEAC) of synthesized compounds L5, L6,** 1,** and** 2** at 1 mM concentration in the FRAP assay compared to the natural precursor boldine (L1), the commercial drug oxaliplatin, and the commercial antioxidant BHT.

The results indicate that 3-carboxyboldine (L5) exhibits a reducing antioxidant power 11 times greater than that of 3-carboxypredicentrine (L6) and 2 times greater activity than that of the natural substrate boldine (L1). Platinum coordinated compound** 1** exhibits an equivalent activity (*P* > 0.05) compared to 3-carboxyboldine (L5), which is approximately 30 times more than the reducing activity of the commercial drug oxaliplatin. However, 3-carboxypredicentrine (L6) exhibits a decreased reducing power compared to the natural product boldine (L1) and the commercial antioxidant BHT. Platinum coordination compound** 2** exhibits approximately 4 times more reducing activity than oxaliplatin. The difference in activity between both of the carboxy derivatives of boldine (L5 and L6) and their platinum compounds (**1** and** 2**) is in agreement with the results obtained in the DPPH assay. The greater reducing antioxidant power of 3-carboxyboldine (L5) may be due to the presence of the phenol at the C-9 position of the aporphinic skeleton.

### 3.5. *In Vitro* Inhibition Growth Assay

The inhibitory concentrations (expressed as IC_50_) of the carboxy derivatives of boldine (L5·HCl and L6·HCl), their corresponding platinum(II) coordination compounds (**1** and** 2**), and oxaliplatin toward two human tumor cell lines (MCF-7 and HT-29) and one human nontumor cell line (CCD-841) are shown in Table 2. No measurable cytotoxic activity (>100 *μ*M) against the studied cell lines was observed for L5 and L6, which is in agreement with the behavior of L6 toward the previously tested MDA-MB-231, PC-3, and DHF cell lines [19]. However, their platinum-derived compounds (**1** and** 2**) exhibited a similar biological activity against the two human tumor cell lines studied and were slightly less cytotoxic (two to four times) against the human nontumor cell line (CCD-841) compared to oxaliplatin. These results revealed that naturally derived oxaliplatin analogues** 1** and** 2** preserve the cytotoxicity featured by platinum-based compounds and the enhanced antioxidant properties of these compounds do not interfere with their cytotoxic activity.

**Figure 2:**
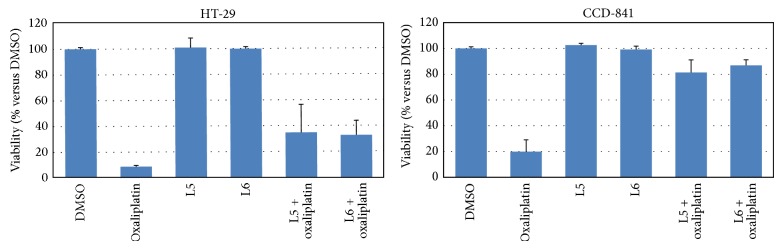
Viability of 24 h pretreated (with 50 *µ*M of L5·HCl or L6·HCl) human tumor colon (HT-29) and human nontumor colon (CCD-841) cell lines prior to oxaliplatin administration (50 *µ*M).

To determine whether carboxy boldine derivatives L5 and L6 contribute to decreasing the cytotoxic activity of the platinum moiety especially in a human nontumor cell line, pretreatment assays were performed. The preadministration of L5·HCl or L6·HCl (50 *µ*M) for 24 h in the colon tumor (HT-29) and colon nontumor (CCD-841) cell lines prior to the oxaliplatin (50 *µ*M) regimen significantly increased the cell viability to approximately 33% for the tumor cell line and approximately 85% for the nontumor cell line (Figure 2). The improved enhancement in cell survival that was observed in the nontumor cell line is a promising result, and the potential biological role exerted by L5 and L6 should be elucidated.

## 4. Conclusions

The synthesis of platinum-based compounds with recognized antioxidant compounds as ligands has not been sufficiently developed as a new approach for the design of a potentially new therapy with lesser secondary toxicities. In this study, we reported the synthesis and characterization of a new oxaliplatin analog [Pt(dach)(L5)] (**1**), where L5 = 3-carboxyboldine, as a continuation of the work on the previously reported [Pt(dach)(L6)] (**2**), where L6 = 3-carboxypredicentrine. L5 was prepared from the natural product boldine (L1) in 19% overall yield in three steps. The antioxidant activity of the natural precursor (L1), synthesized compounds L5, L6,** 1,** and** 2,** and oxaliplatin was investigated using two chemical-based assays. The results indicated that compounds** 1** and** 2** exhibited an antioxidant behavior that was several times higher than oxaliplatin (130 and 13 times for** 1** and** 2** in DPPH assay and 30 and 4 times for** 1** and** 2** in FRAP assay). The difference in the antioxidant activity of synthesized platinum compounds** 1** and** 2** correlated well with the enhanced antioxidant activity of L5 compared to L6. The cytotoxic activity (expressed as IC_50_) of** 1** and** 2** was comparable to the commercial drug oxaliplatin toward the two human tumor cell lines studied and approximately two to four times lower for the human nontumor cell line. In addition, carboxy derivatives L5 and L6 exhibited no measurable activity toward any of the studied cell lines. The pretreatment assay revealed a cytoprotective effect for L5 and L6 against oxaliplatin toxicity, especially on the nontumor cell line. This promising result will be confirmed in other tumor and nontumor cell lines and may promote the incorporation of natural compounds in chemotherapeutics regimes.

## Supplementary Material

1H and 13C NMR spectra of 3-carboxyboldine (L5) are shown. 1H NMR spectrum of [Pt(dach)(L5)] (1) is provided and compared with the previously reported for [Pt(dach)(L6)] (2). Infrared spectra of 3-carboxyboldine (L5) and [Pt(dach)(L5)] are also included.

## Figures and Tables

**Table 1:** Antioxidant activity (IC_50_) in DPPH assay.

Compound	IC_50_ (mM)
L1	1.71 ± 0.03
L5	3.20 ± 0.10
L6	58 ± 8
**1**	2.68 ± 0.08
**2**	26 ± 9
Oxaliplatin	350 ± 70
Trolox	0.40 ± 0.01

**Table 2:** Inhibitory concentration (IC_50_) of compounds L5, L6, **1,** and **2** compared to oxaliplatin toward two human tumor cell lines (MCF-7 and HT-29) and one nontumor cell line (CCD-841).

Compound	IC_50_ (*μ*M)		
	MCF-7	HT-29	CCD-841
L5	>100	>100	>100
L6	>100	>100	>100
**1**	2.8 ± 0.5	15.1 ± 2.1	11.3 ± 0.4
**2**	4.1 ± 0.4	5.9 ± 1.2	19.1 ± 2.0
Oxaliplatin	5.8 ± 0.8	7.7 ± 1.2	4.7 ± 0.8
