# Supplementary material for: Oxaliplatin Analogues with Carboxy Derivatives of Boldine with Enhanced Antioxidant Activity
Source: Bioinorg Chem Appl. 2015 Feb 28;2015:920143. doi: 10.1155/2015/920143 (PMC4359857; doi:10.1155/2015/920143)

==
Supplementary Material to be published electronically
===

Oxaliplatin analogues with carboxy derivatives of boldine with enhanced antioxidant activity.

Marco Mellado[a], Carlos Jara[c], David Astudillo[b], Joan Villena[b], Patricio G. Reveco[a] and Franz A. Thomet[a*]

[a] Department of Chemistry, Universidad Técnica Federico Santa María, Avenida España N° 1680, Valparaíso, Chile.

[b] School of Medicine, Centro de Investigaciones Biomédicas (CIB), Universidad de Valparaíso, Avenida Hontaneda N° 2664, Valparaíso, Chile.

[c] School of Medicine, Laboratorio de Investigación – Estrés Oxidativo, Universidad de Valparaíso, Avenida Brasil N° 1560, Valparaíso, Chile.

Contents

		Page. No.
1.	Figure 3: ^{1}H-NMR spectrum of 3-carboxyboldine (L5)	2
2.	Figure 4: ^{13}C-NMR spectrum of 3-carboxyboldine (L5)	3
3.	Figure 5: ^{1}H-NMR spectra of [Pt(dach)(L5)] (**1**) and [Pt(dach)(L6)] (**2**)	4
4.	Figure 6: Infrared spectrum of 3-carboxyboldine (L5)	5
5.	Figure 7: Infrared spectrum of [Pt(dach)(L5)] (**1**)	5

Figure 3: ^{1}H-NMR spectrum of 3-carboxyboldine (L5) in DMSO-d_6.

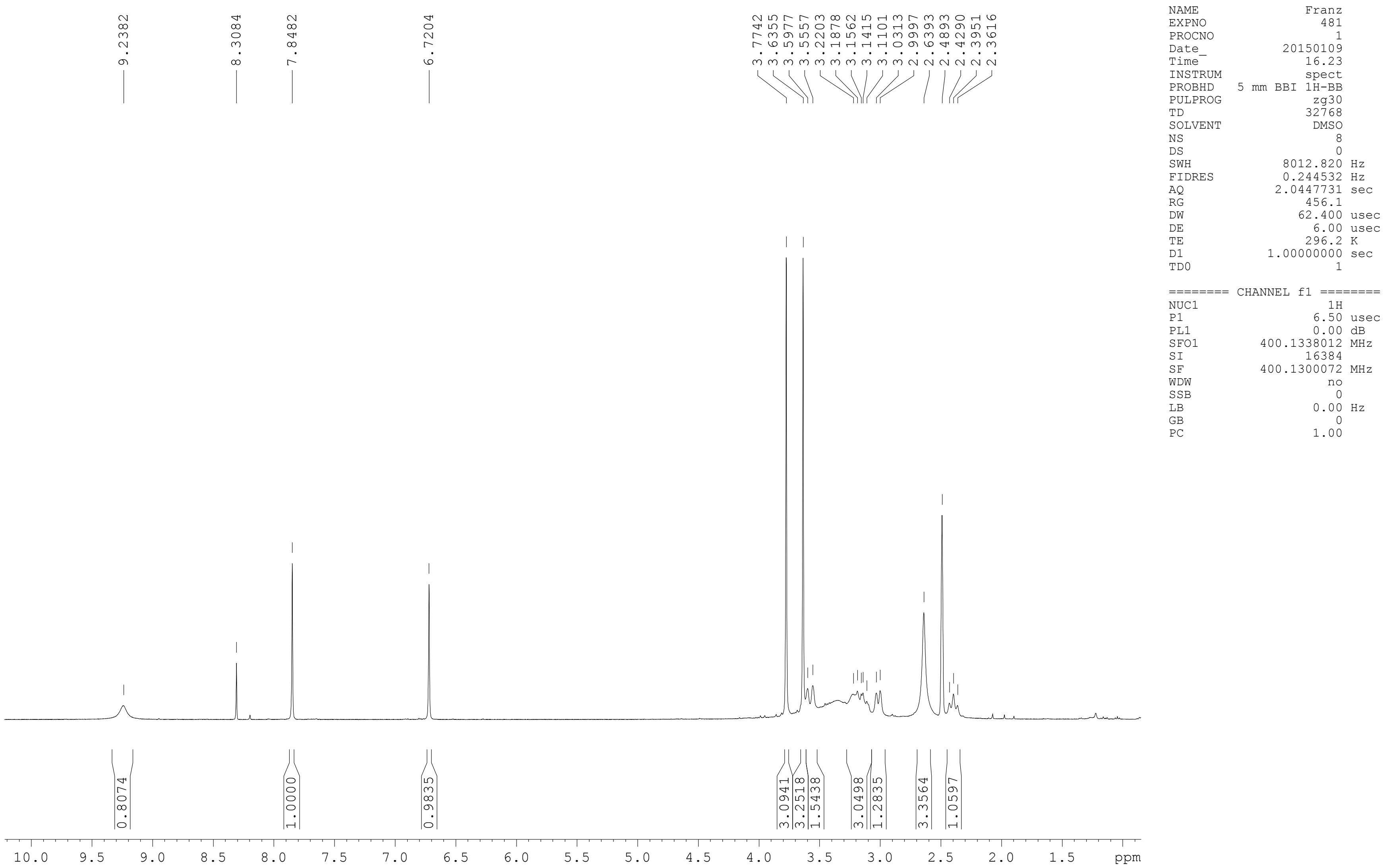

Figure 4: ^{13}C-NMR spectrum of 3-carboxyboldine (L5) in DMSO-d_6.

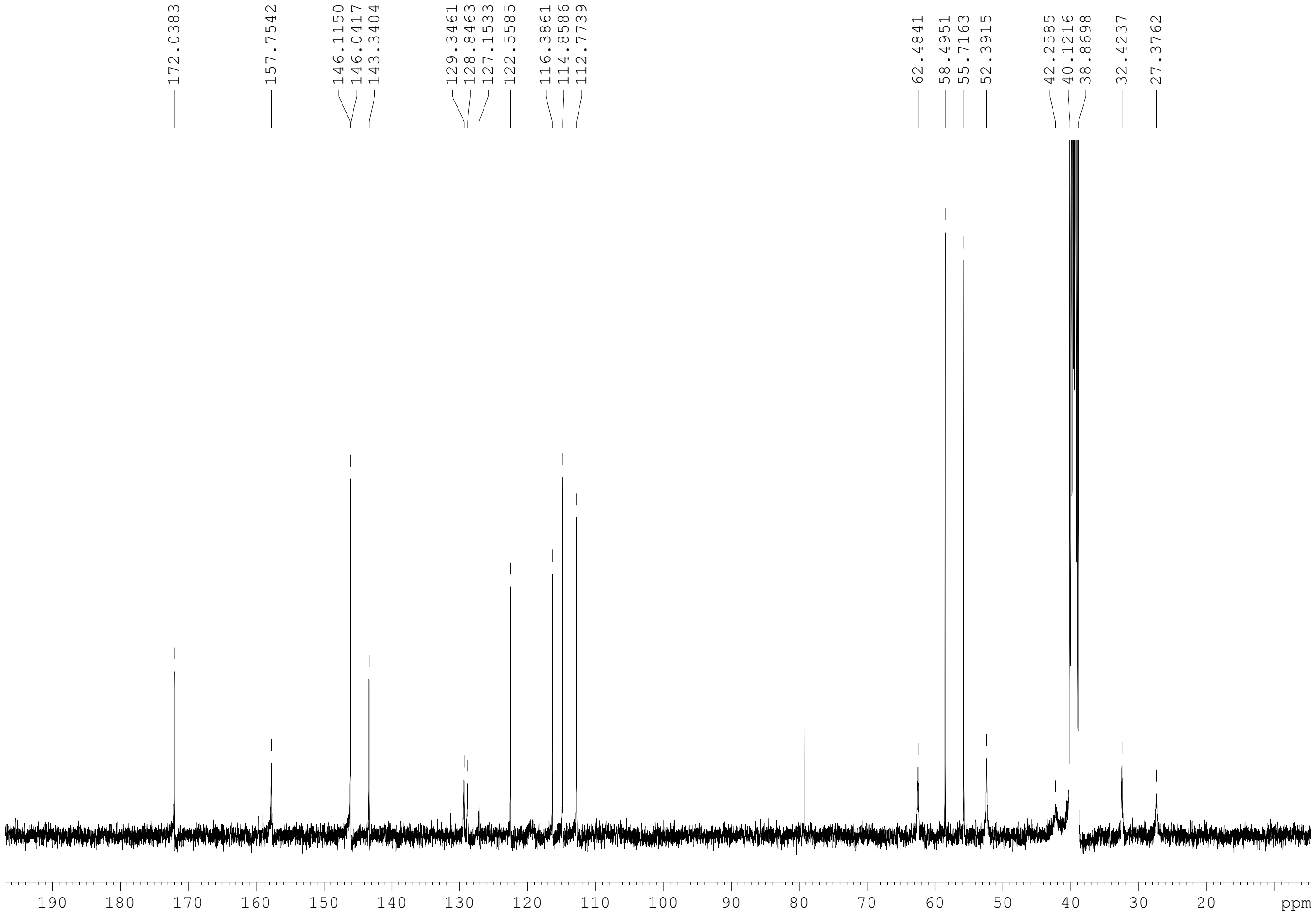

NAME	Franz
EXPNO	482
PROCNO	1
Date_	20150110
Time	4.42
INSTRUM	spect
PROBHD	5 mm BBI 1H-BB
PULPROG	zgpg30
TD	32768
SOLVENT	DMSO
NS	16000
DS	0
SWH	23980.814 Hz
FIDRES	0.731836 Hz
AQ	0.6832628 sec
RG	512
DW	20.850 usec
DE	6.00 usec
TE	298.2 K
D1	2.00000000 sec
D11	0.03000000 sec
TD0	1
======== CHANNEL f1 ========	
NUC1	13C
P1	14.00 usec
PL1	-6.00 dB
SFO1	100.6242995 MHz
======== CHANNEL f2 ========	
CPDPRG2	waltz16
NUC2	1H
PCPD2	85.00 usec
PL2	6.00 dB
PL12	23.00 dB
PL13	23.00 dB
SFO2	400.1316005 MHz
SI	32768
SF	100.6128203 MHz
WDW	EM
SSB	0
LB	1.00 Hz
GB	0
PC	1.40

Figure 5: ^{1}H-NMR spectra of [Pt(dach)(L5)] (**1**, top) and [Pt(dach)(L6)] (**2**, bottom) in DMSO-d_6.

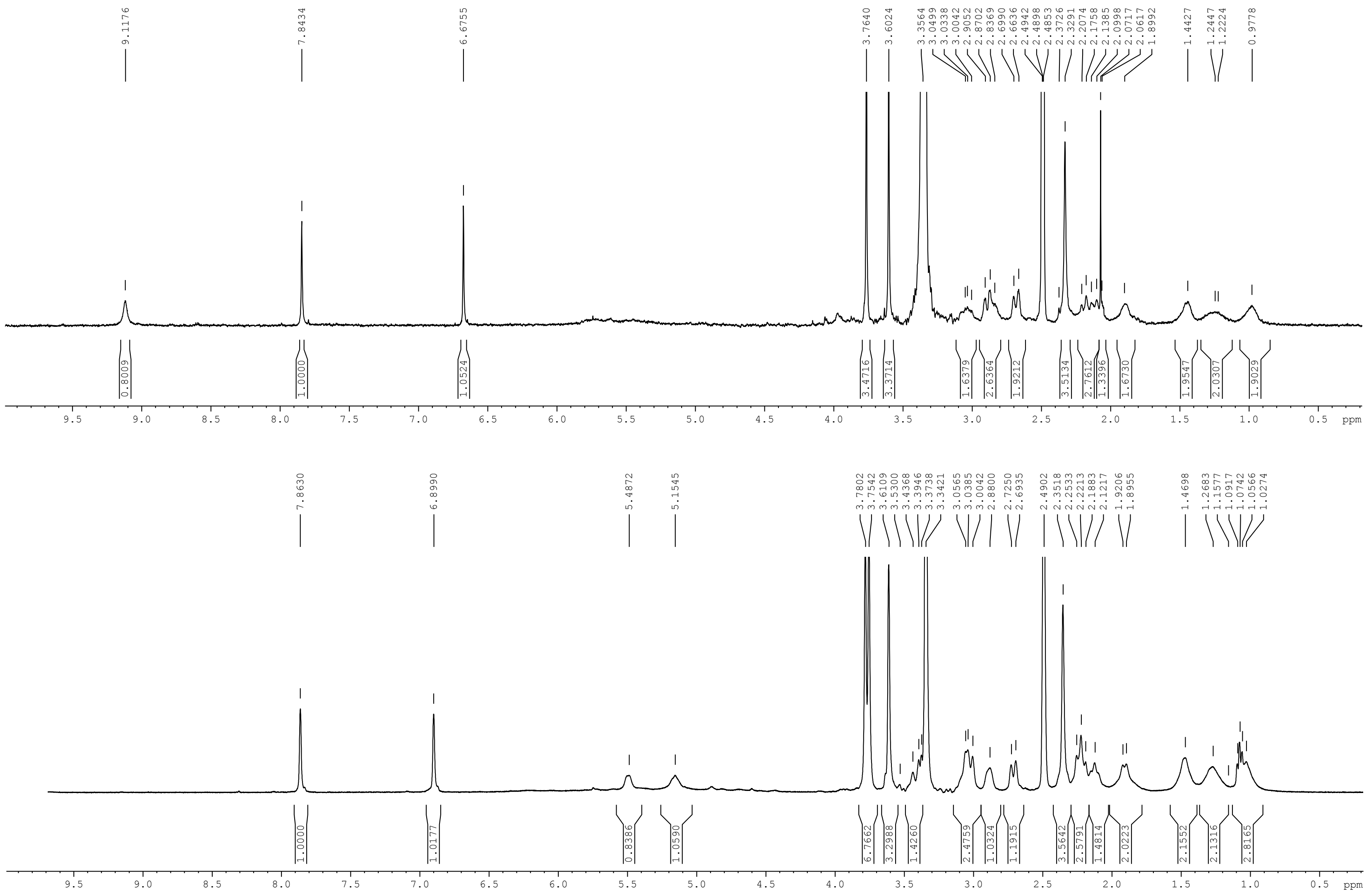

Figure 6: Infrared spectrum of 3-carboxyboldine (L5).

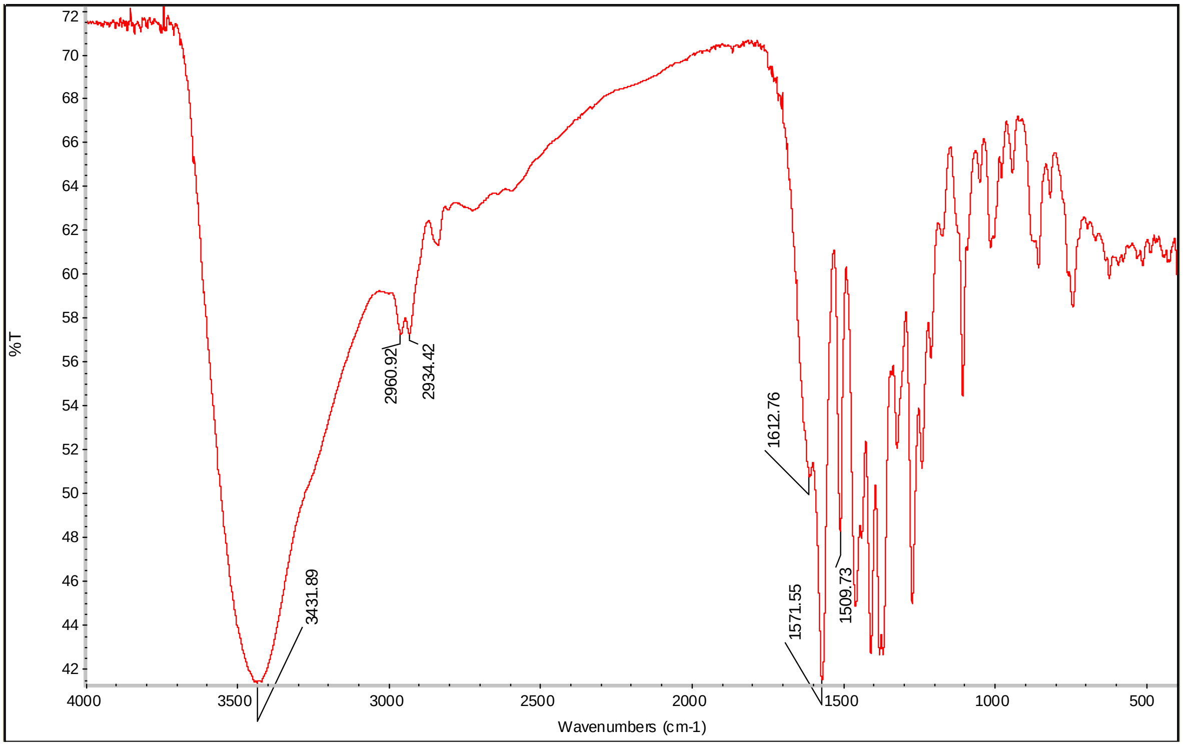

Figure 7: Infrared spectrum of [Pt(dach)(L5)] (**1**).

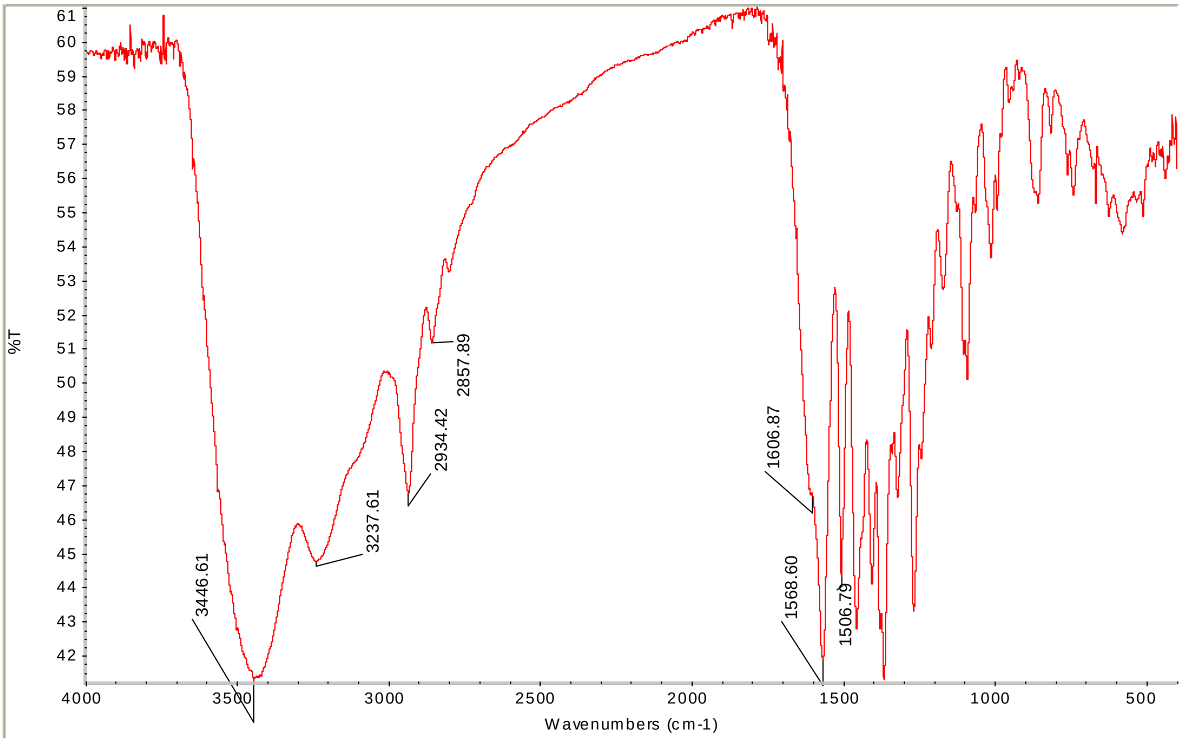

Supplement: Supplementary file 1 — 1H and 13C NMR spectra of 3-carboxyboldine (L5) are shown. 1H NMR spectrum of [Pt(dach)(L5)] (1) is provided and compared with the previously reported for [Pt(dach)(L6)] (2). Infrared spectra of 3-carboxyboldine (L5) and [Pt(dach)(L5)] are also included. [file 920143.f1.pdf]
